# A history of wearing a face mask in ancient Iran

**DOI:** 10.1186/s40001-023-01120-8

**Published:** 2023-04-21

**Authors:** Amir Hossein Jamshidi, Hamed Arezaei, Bahareh Sadat Yousefsani, Majid Dadmehr

**Affiliations:** 1 Department of Traditional Pharmacy, School of Persian Medicine, Iran University of Medical Sciences, Tehran, Iran; 2 Institute for Studies in Medical History, Persian and Complementary Medicine, Iran University of Medical Sciences, Tehran, Iran; 3 Department of Traditional Medicine, School of Persian Medicine, Iran University of Medical Sciences, No 847, Behesht St. South Side of Shahr Park, Vahdat-e-Islami St, Hasan Abad Sq, Tehran, 1114733311 Iran

Dear Editor,

We read with great interest the article by Matuschek et al. entitled “The history and value of face masks” [1]. The authors well mentioned a history of wearing a face mask since ancient time, however, we suggest some more details about this topic which can be interesting and complementary.

During the COVID-19 pandemic, the World Health Organization (WHO) strongly recommended to citizens of all countries to wear a face mask as part of a general strategy to control the transmission and save human lives, in addition to adoption social distancing and hand hygiene [2]. This was not the first time that community members needed to face masks for protection. Covering the nose and mouth has traditionally been practiced in ancient societies for a variety of purposes, including hygiene measures against infectious diseases, showing or hiding, fleeing or fighting, protecting or punishing [3, 4]. In the mid-fourteenth century plague epidemic in Europe, medieval physicians known as ‘plague doctors’, wore clothing that included a beaked white mask, black hat, and waxed gown to keep safe them against the plague or black death. The beak was also full of plants, with the possibility that they could absorb harmful air [1, 4]. Most recently, several studies have scrutinized the history of the face mask wearing in ancient civilizations from different aspects, nevertheless, none of them have mentioned the use of mask in ancient Iran [1, 3, 4]. Reviewing historical evidence shows that before the Renaissance, Zoroastrian priests in Persia covered their noses and mouths with veils similar to face mask objects for medical and none medical purposes.

From the beginning of the Achaemenid dynasty in 553 B.C. to the end of the Sassanid dynasty in 651 C.E. Avestan or Zoroastrian medicine was common in ancient Iran. It was originally derived through the holy book of Zoroastrians named Avesta. It is believed that the prevention of mental and physical illnesses was of particular importance [5, 6]. ‘*Vendīdād’* one of the major divisions of this book includes various topics in the field of medical history, rules governing medical practice, and guidelines for health care and hygiene [5]. Several types of healer-physicians, which selected from Zoroastrian priests such as surgeons, physicians who worked with herbal medicines, and physicians who treated with holy words are named in this book [5, 6]. They used face masks called ‘*Panām*’ in front of their noses and mouths to prevent contaminating others. In the old Persian language, ‘*Panām*’ means a holder against something. ‘*Panām*’ is two pieces of white rectangular cotton cloth, which are tied on the face two knuckles below the mouth with two ribbons tied behind the head. It was also named the veil ‘*Rūband*’, which was worn by Zoroastrian priests ‘*Mūbads*’ as a face covering in religious ceremony (Fig. 1). They believed that the use of ‘*Panām*’ was intended to prevent the breath and vapor of a person's mouth from reaching the sacred element and also show respect [7].

**Fig. 1:**
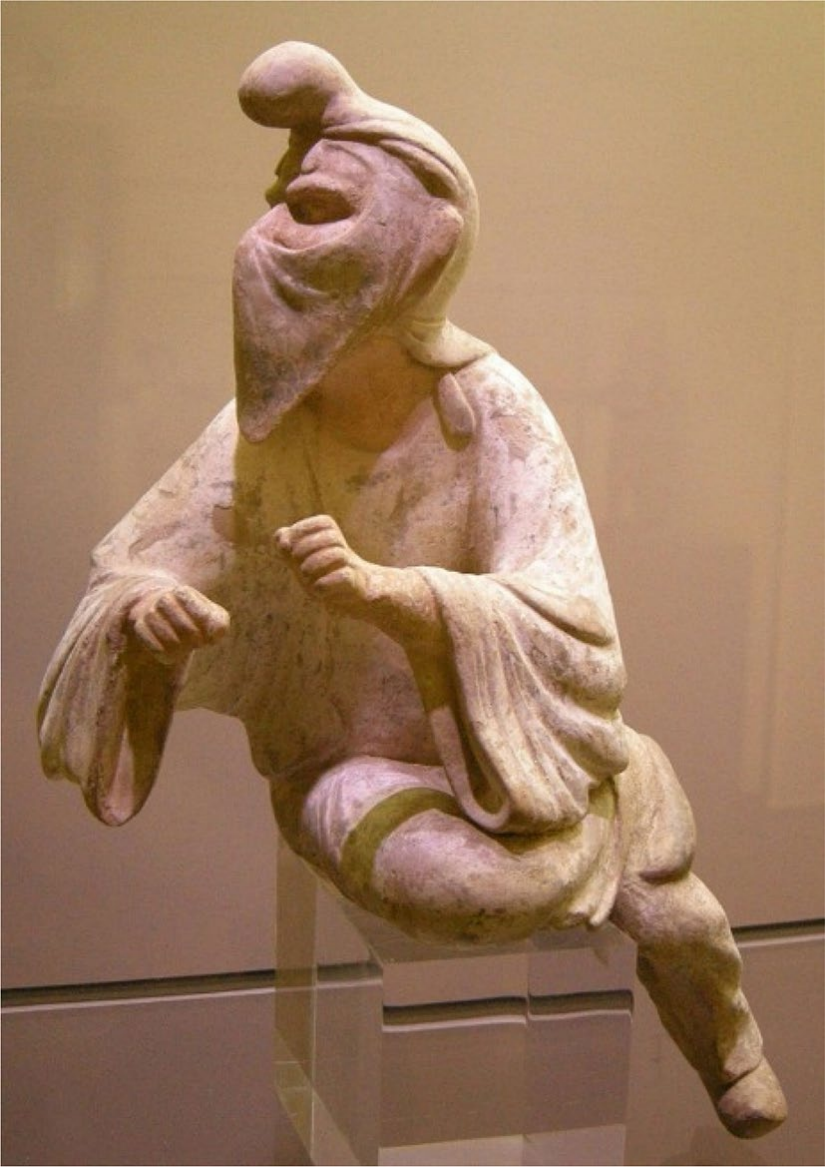
A Zoroastrian priest who wearing a ‘*Panām*’. The statue is housed in the Museum of Oriental Art in Turin, Italy

During the pre-Islamic period, scientists of ancient Iran have been considered the term contagious and the theory of prevention. They divided physical illnesses into two categories, contagious and non-contagious. In the ‘*Vendīdād*’, contagious diseases were called ‘*Pauru-mahrka*’ meaning deadly or very deadly. Physicians of that period were well aware that some diseases were epidemic and could be transmitted from person to person, therefore, after visiting one patient, they cleaned themselves thoroughly and considering a time interval, visited another patient to prevent transmission of the disease. Moreover, they wore ‘*Panām*’ and covered their face from the nose to under the chin to prevent the spread of disease. The physicians also kept the patient out of the community for some time so as not to infect others [8].

There is another example that shows ancient Iranians were familiar with the concept of transmission and the effect of wearing a face mask in preventing disease transmission. *Al-Jāḥiẓ* (776–869 AD)—a prose writer and author of works of literature—in his book ‘*Kitāb al-Ḥayawān*’ mentioned: “Iranians in the southern regions, in dealing with people who died from scorpion stings and their bodies stink, covered their noses and mouths with ‘*Panām*’ for fear of transmission, and only faced the corpse by wearing a face mask” [9].

## Data Availability

All data and materials can be accessed via MD and HA.

## References

[1] Matuschek C, Moll F, Fangerau H, Fischer JC, Zänker K, Van Griensven M (2020). The history and value of face masks. Eur J Med Res.

[2] Liao M, Liu H, Wang X, Hu X, Huang Y, Liu X (2021). A technical review of face mask wearing in preventing respiratory COVID-19 transmission. Curr Opin Colloid Interface Sci.

[3] Strasser BJ, Schlich T (2020). A history of the medical mask and the rise of throwaway culture. Lancet.

[4] Isaacs D (2021). Mask wearing: a historical, cultural and ethical perspective. J Paediatr Child Health.

[5] Zargaran A, Mehdizadeh A, Yarmohammadi H, Mohagheghzadeh A (2012). Zoroastrian priests: ancient Persian psychiatrists. Am J Psychiatry.

[6] Nayernouri T (2015). a brief history of ancient Iranian medicine. Arch Iran Med.

[7] https://www.iranicaonline.org/articles/clothing-xxvii. Accessed 12 Feb 2021.

[8] KavianiPouya H (2014). Medical history of ancient Iran.

[9] Al-Jāḥiẓ (AbūʿUthman ʿAmr ibn Baḥr al-Kinānī al-Baṣrī). Kitāb al-Ḥayawān (The book of living). Beirut: Dar al Kotob al Ilmiyah; 2004, vol 4, p 73.

